# Molecular archeological evidence in support of the repeated loss of a papillomavirus gene

**DOI:** 10.1038/srep33028

**Published:** 2016-09-08

**Authors:** Koenraad Van Doorslaer, Alison A. McBride

**Affiliations:** 1 Lab of Viral Diseases, NIAID, NIH, Bethesda, MD, USA

## Abstract

It is becoming clear that, in addition to gene gain, the loss of genes may be an important evolutionary mechanism for many organisms. However, gene loss is often associated with an increased mutation rate, thus quickly erasing evidence from the genome. The analysis of evolutionarily related sequences can provide empirical evidence for gene loss events. This paper analyzes the sequences of over 300 genetically distinct papillomaviruses and provides evidence for a role of gene loss during the evolution of certain papillomavirus genomes. Phylogenetic analysis suggests that the viral E6 gene was lost at least twice. Despite belonging to distant papillomaviral genera, these viruses lacking a canonical E6 protein may potentially encode a highly hydrophobic protein from an overlapping open reading frame, which we designate E10. Evolutionary pressure working on this alternative frame, may explain why, despite having lost the E6 open reading frame between 20 and 60 million years ago, evidence of an E6-like protein is conserved.

Compared to most organisms, viral genomes tend to be small, yet packed with coding sequences. Many viruses are able to limit the size of their genome by coding for proteins transcribed from overlapping open reading frames (ORFs), by utilizing alternative splicing, and by minimizing intergenic regions. There is a tendency to think that evolution increases the genetic complexity of an organism^1^. However, it has been suggested that adaptive gene losses may provide organisms with distinctive evolutionary advantages^2^. For example, it has been suggested that the reduction in the number of viral genes may be important during poxvirus speciation and environmental (host tissue) niche selection^3^.

The study of gene loss is complicated by the fact that once a gene acquires an inactivating mutation the forces of natural selection no longer limit the mutation rate. Thus, with time, the molecular scar resulting from gene loss will fade from the genome. It has been estimated that once a gene is lost, evidence of adaptive evolution persists only for about 250,000 years^4^,^5^,^6^. However, comparative genomics has been useful to identify evidence of ancient gene loss events^1^.

Papillomaviruses have been described to infect a wide array of vertebrates^7^, and most hosts are likely infected with an extensive, highly species-specific repertoire of viruses. To date, over 300 individual papillomavirus types have been completely characterized at the molecular level^8^. In addition to a core set of five viral proteins, many papillomaviruses encode for proteins (E5, E6, and E7) involved in modulating the cellular environment^9^,^10^,^11^,^12^. In the oncogenic viruses these proteins have been shown to be potent oncogenes. Despite the fact that these proteins are thought to be essential for the viral life cycle, comparative genomic analysis suggested that some viruses do not code for these “accessory” genes^13^.

In the present manuscript we have applied “molecular archeology” on this treasure-trove of complete genomic data to provide evidence for gene loss throughout papillomaviral evolution.

## Results and Discussion

All known papillomaviruses code for at least four conserved core proteins (E1, E2, L1 and L2). Together, the L1 and L2 proteins make up the viral capsid^14^,^15^, while the E1 and E2 are key modulators of replication and transcription^16^,^17^. The L1 ORF is also the basis for the current classification system. Based on genetic similarity across the L1 ORF, papillomavirus types are classified into genera designated by Greek letters. Genera are further divided into viral species^18^,^19^. Located downstream of the L1 ORF, all papillomaviruses contain an untranslated long control region (LCR)^20^, which is usually followed by the E6 and E7 open reading frames. Papillomavirus infection drives cell proliferation that may lead to oral, genital, or skin tumors. The induction of cell proliferation has been related mainly to the expression of two small oncoproteins, E6 and E7^9^. During a productive infection, the expression of E6 and E7 is tightly regulated, and in the cancer-associated viruses, these proteins have been shown to be potent oncogenes^10^,^11^. Located between the E2 and L2 ORFs, some viruses encode a short, hydrophobic, membrane-associated proteins known as E5^12^,^21^. In addition, certain papillomaviruses (i.e. the members of the Xi and Kappa-papillomavirus genera) encode a highly hydrophobic protein in the genomic location typically reserved for E6^12^. To avoid confusion with the well-defined E5 located between E2 and L2, we propose to rename this upstream ORF E10. Despite not being evolutionary related, some of these E5 and E10 proteins have been shown to have transforming activity *in vitro*. However their roles during productive infection are less defined^12^.

The E6 protein of most (mammalian) papillomaviruses contains two highly conserved 70-residue zinc-binding repeats. This ability to bind zinc has been shown to be essential for their functionality^22^. It has been suggested that the extant mammalian E6 protein is the result of an ancestral duplication of a single zinc binding motif containing E6. These single domain E6 proteins are still present in extant bird and turtle viruses^13^,^23^,^24^.

**Figure 1:**
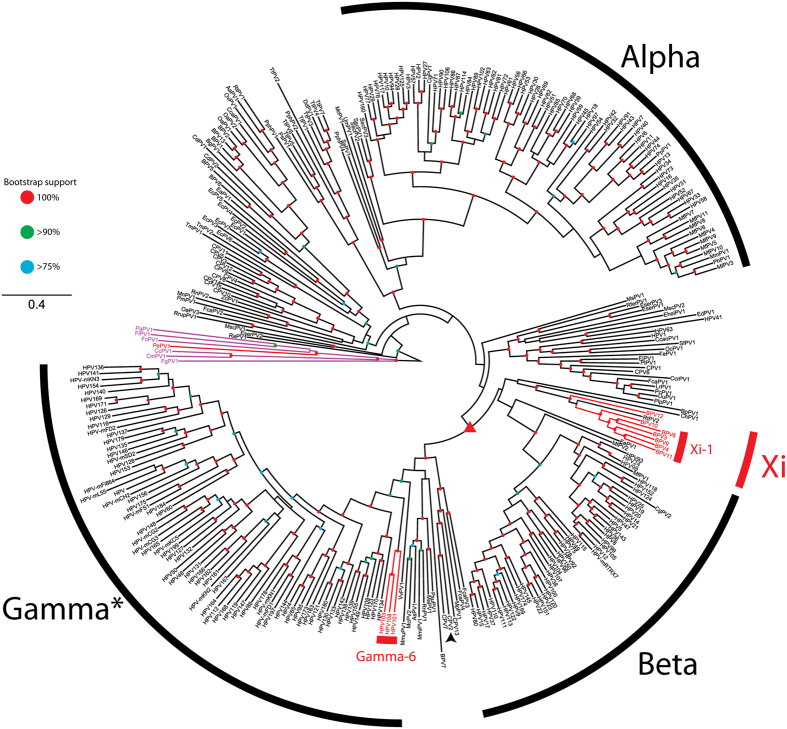
Maximum likelihood phylogenetic tree representing the evolutionary relationships among the *Papillomaviridae*. Maximum likelihood phylogenetic tree of the *Papillomaviridae*. This tree is based on a partitioned supermatrix derived from the E1, E2, L2 and L1 nucleotide sequences. Viruses indicated with a red clade do not contain a canonical E6 ORF, purple clades identify the avian/turtle viruses that contain a “single domain” E6 protein. Classification was based on^18^,^19^. The asterisk (*) indicates that some members of this clade have not yet been officially recognized as members of the Gammapapillomavirus genus. Colored nodes indicate bootstrap support (red = 100%, green >90%, blue >75%). The red triangle indicates the MRCA of Xi- and Gamma papillomaviruses. The arrowhead highlights CPV2, which was chosen to root the phylogenetic tree in Fig. 2.

It has been proposed that E6’s main function may be to neutralize some of the unwanted consequences of E7 expression^10^. However, not all papillomaviruses contain a canonical E6 ORF (Fig. 1 and^13^). Figure 1 summarizes the evolutionary history of the different papillomavirus genera, with the presence/absence of a canonical E6 mapped; red branches indicate individual papillomaviruses that do not contain a canonical E6 ORF (E6-minus viruses). Purple branches highlight the avian and reptilian papillomaviruses which contain a highly modified, ancestral E6 protein^23^. This analysis illustrates that certain viral clades are apomorphic for the presence/absence of E6. In other words, certain members of the Xi- and Gammapapillomavirus genera do not contain a canonical E6 ORF (E6-minus). The position of these E6-minus viruses in separate clades indicates that the E6 gene has been lost at least twice throughout papillomavirus evolution. An alternative, less likely explanation is that the most recent common ancestor (MRCA; indicated with triangle in Fig. 1) did not encode for an E6 ORF, with extant E6 proteins being the result of several convergent evolutionary events.

The genus Xipapillomavirus groups viruses infecting even-toe ungulates, while most viruses in this genus do not contain a canonical E6 ORF, they encode a hydrophobic protein (E10; formerly known as E8) of about 40 amino acids in the same genomic position^12^. In the case of BPV4 E10, this protein has been implicated in the development ofalimentary tract carcinomas^25^. Interestingly, RtPV2 a Xipapillomavirus isolated from reindeer contains an E6 ORF with an embedded E10 ORF in the +1 frame. Given the position of RtPV2 in the phylogenetic tree, this suggests that, within the genus Xipapillomavirus, E6 was lost after the acquisition of E10. Furthermore, the available data suggests that E6 may have been lost two separate times within this clade. A first loss occurred along the clade leading to BPV12, while a second loss occurred following the divergence of RtPV2 and the Xi-1 species (BPV3, −4, −6, −9, −10, and −11; Fig. 1). Alternatively, it is possible that the concatenated gene tree does not accurately describe the evolutionary history of the Xipapillomavirus genus, and that RtPV2 is in fact basal to the remaining members of the Xipapillomavirus genus. While the evolutionary history of papillomaviruses is complicated, papillomaviruses have been shown to have coevolved with their hosts^26^. Since *Bos taurus* and *Rangifer tarandus* diverged roughly 35 million years ago (MYA)^27^, the above mentioned second loss must have occurred in sync with the host divergence event, implying that this loss must have occurred over 35 MYA.

**Figure 2:**
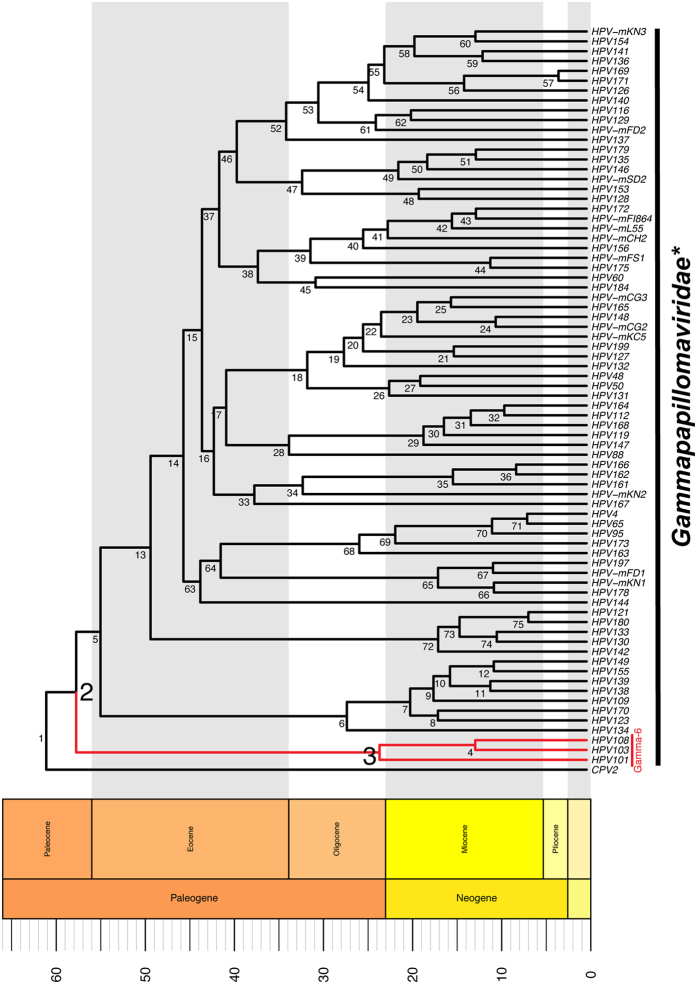
Time calibrated phylogenetic tree of Gammapapillomavirus sequences. The evolutionary rate estimated for feline PVs^26^ was used to estimate the divergence time of the indicated nodes. Classification was based on^18^,^19^. The asterisk (*) indicates that some members of this clade have not yet been officially recognized as members of the Gammapapilliomavirus genus. Support values and exact estimates (with 95% highest posterior density) for each node are presented in supplementary Table 2. The Gamma-6 species is highlighted in red. The tree and geological column were generated using the APE^52^, phyloch (available from http://www.christophheibl.de/Rpackages.html), and strap^53^ packages within R. The scale bar indicates millions of years before the present. The Gammapapillomavirus genus tree was rooted on CPV2 (indicated by arrowhead in Fig. 1).

The members of the Gamma-6 species (HPV101, HPV103 and HPV108) do not encode a canonical E6 gene. Since all the other known members of the *Gammapapillomavirus* genus do contain such a gene, this suggests that the gene loss occurred in the most recent common ancestor of the Gamma-6 species. Likewise, this genetic event must have occurred after the Gamma-6 viruses diverged from the other members of the *Gammapapillomavirus* genus. Following the acquisition of an inactivating mutation, the lack of evolutionary selection will be manifested by the functional gene getting erased from the genome. Therefore, evidence for gene-loss can only be detected if viral species diverged in the relatively recent past. The evolutionary rate for papillomavirus has been estimated to be around 5–10 times faster than that of the host^26^. These published rate estimates were used to calculate the divergence times of members of the *Gammapapillomavirus* clade (Fig. 2). This analysis indicates that the gamma-6 species last shared a common ancestor with other *Gammpapillomaviruses* around 60 million years ago (node 2 in Fig. 2, and supplementary Table 1), while the MRCA of the current Gamma-6 viruses speciated around 23.4 MYA (node 3 in Fig. 2, and supplementary Table 2). Therefore, we hypothesize that the E6 ORF was lost from the Gamma-6 species between 20 and 60 MYA ago. This implies that the loss of E6 occurred following the emergence of the *Simiiformes*, but prior to the speciation event that gave rise to the *Hominoidea* (81 MYA to 20 MYA, respectively^28^). This implies that E6-minus viruses may infect both old-world and new-world monkeys. The isolation of more primate E6-minus viruses will be required to improve on these estimates, and confirm the presence of E6-minus viruses in new-world monkeys. The loss of the E6 protein from the Gamma-6 species was previously estimated to have occurred between 15–30 MYA^29^. This estimate was based on the L1 ORF of nine evolutionary highly divergent papillomaviruses (including an avian papillomavirus). Chen and colleagues estimated the divergence between the avian and mammalian viruses to have occurred around 50 MYA, while the respective hosts likely diverged about 300 MYA ago^30^. It is likely that the limited number of highly divergent sequences used resulted in an underestimation of the real evolutionary history of these ancient viruses. Furthermore, the evolution of papillomaviruses likely included ancient duplication events, further complicating analysis over long evolutionary distances^13^,^31^.

**Figure 3:**
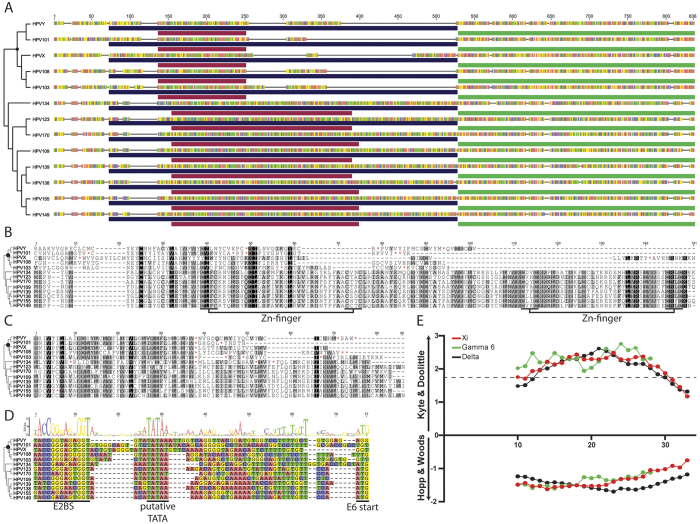
Viruses within the Gammapapillomavirus 6 species contain a putative E5 like open reading frame. (**A**) Evidence of a C-terminal deletion within the E6 protein of the Gamma-6 viruses. Alignment of the sequence from the promoter proximal E2 binding site to the stop codon of E7 extracted from Gamma-6 and Gamma-7 papillomaviruses. The phylogenetic tree indicates the evolutionary history, with the black circle highlighting the Gamma-6 species MRCA. Colors indicate nucleotides (A = red; T = green, C = blue, G = yellow). Supplemental Figure 1 has a base pair level version of this alignment. The purple bar indicates the E6 ORF (alignment in 3B), red and green bars mark the putative E10 and E7 ORFs (E10 alignment in 3C). (**B**) Sequence alignment of the putative gamma-6 E6 remnant and related Gammapapillomavirus E6 proteins. The alignment is shaded by conservation. Red asterisks (*) indicate internal STOP codons. Zinc-binding motifs are indicated below the alignment. The phylogenetic tree indicates the evolutionary history, with the black circle highlighting the Gamma-6 species MRCA. (**C**) Sequence alignment of the putative gamma-6 E10 protein and related Gammapapillomavirus proteins. The alignment is shaded by conservation. Red asterisks (*) indicate internal STOP codons The phylogenetic tree indicates the evolutionary history, with the black circle highlighting the Gamma-6 species MRCA. (**D**) The putative promoter of E6-minus viruses is located in the correct syntenic environment. Sequence alignment of the DNA sequence upstream of the putative E6 start codon. A consensus E2 binding site, putative TATA box, and E6 start codon are indicated. A sequence logo shows the consensus sequence. The phylogenetic tree indicates the evolutionary history, with the black circle highlighting the MRCA of the Gamma-6 species. (**E**) Hydropathicity scale of the Delta E5, Xi E10, and Gamma-6 E10-like proteins. Kyte – Doolittle^49^ and Hopp – Woods^50^ protein scales were calculated using a sliding window with size of 19 aa. Values were calculated for individual E5-like proteins (Delta; n = 13, Xi; n = 9, and Gamma-6; n = 5). The graph shows the average value for each group. The first 24 windows for the Delta E5 proteins are shown. For the Kyte – Doolittle graphs, values greater than 1.8 (indicated by the dotted line) represent possible transmembrane regions.

The papillomavirus genome organization is highly conserved throughout the roughly 300 million years of viral evolution. The viral early promoter is located within the upstream-regulatory region and transcribes the viral early genes. In order to represent a true gene-loss event, it is essential that the upstream and downstream elements are present, and conserved. When present, the E6 ORF is located upstream of the viral E7 ORF (Fig. 3A). Among members of the *Gammapapillomaviridae*, the E6 ORF is on average 435 base pairs in length (standard deviation of 33). The DNA sequence corresponding to 500 base pairs upstream of the E7 start codon was extracted from HPV101, HPV103 and HPV108. During this process, a literature search turned up evidence for two novel E6-minus viruses (HPV-X and HPV-Y) tentatively belonging to the Gamma-6 species^32^. The upstream DNA sequence was translated in three forward frames and manually explored for the presence of E6 like proteins. The resulting protein sequences were aligned to the E6 protein sequence of some of the gamma-6 viruses’ closest relatives. This alignment is shown in Fig. 3B. While this region acquired many mutations, landmarks of a Gammapapillomavirus E6 protein can be observed. Importantly, while the sequence upstream of position 60 is reasonably well conserved, the downstream sequence has been deleted (Fig. 3A).

The presence of a conserved E2 binding site and TATA box upstream of the putative E6-minus sequence (Fig. 3D) provide further support for the notion that the E6-minus viruses lost an E6 ORF at some point in their evolutionary history.

Despite the fact that E6 was likely lost between 20–60 MYA, an evolutionary scar can still be detected. Notwithstanding the relatively slower evolutionary rate, this is significantly longer than expected. In addition, while the first 60 amino acids of the E6 remnant are fairly well conserved (Fig. 2B), the downstream sequence is highly variable and dotted with indels and stop codons. Interestingly, Gamma-7 viruses contain a highly conserved ORF in the +1 frame of their E6 protein (Fig. 3A,C). Similarly, the E6-minus viruses contain a conserved, yet shorter ORF in this alternative frame. This putative protein is 37 amino acids long and contains many hydrophobic residues, reminiscent of the Xi E10 protein. To further examine the potential similarity between the Gamma-6 and Xi E10-like proteins, two distinct hydrophocity protein scales were calculated (Fig. 3E). Based on these protein scales, the putative gamma-6 E10 protein is predicted to have a hydrophobicity profile similar to the E10 and E5 proteins typically found in members of the Xi and Delta papillomavirus genera respectively (Fig. 3E). The genomic location of this alternative ORF further strengthens the similarity with the Xi papillomavirus E10 protein. Notably, this E10-like protein overlaps with the more conserved portion of the E6 scar (amino acid 25 to 60 in Fig. 3B; nucleotides 137 to 250 in Fig. 3A and supplemental Figure 1). This suggests that selective pressure aimed at maintaining the E10-like protein, may also work to conserve the E6 scar. Furthermore, this implies that, similar to the Xi-genus viruses, Gamma E6-minus viruses may have acquired a hydrophobic, E10-like protein prior to the loss of E6. Interestingly, HPV134, a virus at the root of the Gamma-7 species, does not have the ability to express a putative E10 protein, but expresses a canonical E6 protein, suggesting that either protein may be sufficient for the viral lifecycle. It is tempting to speculate that Gamma-6 E10-like proteins may be expressed and functional, and that this could explain its relatively high level of conservancy.

Most members of the *Gammapapillomavirus* genus were isolated from the skin and oral mucosa^19^,^33^. However, the E6-minus viruses were isolated from cells taken from normal (HPV103), low-grade (HPV108), and high-grade (HPV101) cervical lesions^29^,^34^. Similarly, the HPV-X and HPV-Y viruses were isolated from cervical samples^32^. Suggesting that these E6-minus viruses may have arisen after the adaptation to the cervical milieu. The observation that the E7 protein of gamma-6 viruses induces a phenotype qualitatively similar to that described for the Alphapapillomavirus E7 oncoproteins^34^ further strengthens the idea that these viruses adapted to the mucosal niche. Interestingly, these viruses have a dinucleotide signature which is remarkably similar to other mucosal viruses in the Alphapapillomavirus genus^35^, suggesting a role for convergent evolution following niche adaptation.

The data presented in the present manuscript expands on our understanding of the E6 protein. It appears that an ancestral virus acquired a “single domain E6 protein”. Through duplication, this protein gave rise to the extant two domain E6 protein present in most mammalian viruses. It appears that throughout the evolution of the *Papillomaviridae*, E6 was lost at least twice. Interestingly, loss of E6 may need to be complemented by another protein. The ungulate Xipapillomaviruses evolved an unrelated E10 protein which has been implicated in alimentary tract carcinomas^12^. Similarly, the Gamma-6 viruses acquired an E10-like protein through convergent evolution. In addition to E6-minus viruses, a subset of viruses without an E7 have been described (i.e. viruses belonging to the Upsilon-, Omikron-, Omega-, Dyopi-, and Dyodeltapapillomavirus genera)^13^. Together with the increased divergence rates of these adaptive proteins^20^, it appears that this part of the viral genome may be even more plastic than initially expected^13^,^36^.

It is clear that papillomavirus E6 proteins have evolved to manipulate the cellular environment, thereby allowing for the completion of the viral lifecycle^10^. However, it appears that, at least under certain conditions, the canonical E6 protein is dispensable for the viral lifecycle. Data presented in this manuscript suggests that the loss of E6 may be complemented by the gain of an E10 protein. It is unlikely that E10 will be able to perform all the E6 functions. However, a comparative analysis between both proteins could provide insights into which E6 functions are essential to the viral lifecycle.

In most cases, it is expected that loss of an ORF will be detrimental to the evolutionary success of a virus, yet in certain cases, loss of these ORFs may provide viruses with a distinct evolutionary advantage.

## Materials and Methods

### Source of Sequence data

Sequences were downloaded from the Papillomavirus episteme (PaVE; http://pave.niaid.nih.gov/#home; accessed on 4/1/2015)^8^. The sequences for HPV-x and HPV-y were obtained from the original authors^32^. Papillomaviruses are officially classified into distinct species by the International Committee for the Taxonomy of Viruses (ICTV)^18^. A subset of viruses used in this study have not yet been classified, but were provisionally grouped into specific genera based on taxonomy.

### Phylogenetic tree construction

Sequences for the E1, E2, L1 and L2 open reading frames were aligned at the protein level using the l-INS-I algorithm within mafft^37^,^38^,^39^. Aligned protein sequences were back-translated into nucleotide alignments. The individual alignments were concatenated into a single super matrix. The optimal partition scheme and model of evolution were determined using PartitionFinder (BIC criteria)^40^. The data set was divided into 12 partitions (three codon positions for 4 genes), each partition was estimated to have evolved according to an individual GTR + I + G model^41^,^42^,^43^. RaxML was used to construct the maximum likelihood phylogeny, using the GTRGAMMA model of evolution^44^,^45^. The number of bootstrap replicates (n = 50; Weighted Robinson-Foulds = 1.87) were determined according to the autoMRE criteria implemented in RaxML^46^.

### Dating of the Gammapapillomavirus divergence

The E1, E2, L1 and L2 sequences for a subset of viral sequences (supplementary Table 1) were aligned as described above. The divergence times were estimated using an uncorrelated relaxed clock in BEAST 1.8.2^47^. The partitioning scheme and evolutionary model were the same as in the previous paragraph. While a single tree was estimated, each gene was allowed to evolve according to its own rate. Bayesian MCMC analyses in BEAST were performed using the General time reversal model of evolution allowing gamma-distributed rate heterogeneity among sites. Normal priors were used for evolutionary rate based on the posterior distributions obtained for feline papillomaviruses [mean (stdev); E1: 1.76E-8 (2.8E-9); E2: 2.11E-8 (3.3E-9); L1: 1.84E-8 (2.8E-9); L2: 2.13E-8 (3.3E-9)]^26^. Based on the Maximum Likelihood (ML) analysis, a monophyletic requirement was imposed on the *Gammapapillomaviridae*, the gamma-6 species and the clade excluding the gamma-6 viruses. The MCMC chains were run for 5 × 10E7 generations. Samples were collected every 1000th generation, while a 10% burn-in was discarded. An estimated sample size of >200 was achieved for all parameters. Chain convergence was confirmed using AWTY^48^. Furthermore, the analysis was repeated three separate times after which the results were combined using Logcombiner (version 1.8.2). The reported estimates (Fig. 2 and supplementary Table 2) are based on the combined dataset.

### Protein scale plots

The Kyte – Doolittle^49^ and Hopp – Woods^50^ protein scales were calculated using a custom python script recapitulating the ExPASy ProtScale^51^ output. Briefly, for each point *i* in the graph, the *i* − (*n* − 1)/2 neighboring residues on each side of residue *i* were used to compute the score. The score for residue *i* is the average of the scale values for the amino acids in window with size *n*.

### Data availability

All alignments, scripts, and associated files are available from Figshare (DOI: 10.6084/m9.figshare.3426539).

## Additional Information

**How to cite this article**: Van Doorslaer, K. and McBride, A. A. Molecular archeological evidence in support of the repeated loss of a papillomavirus gene. *Sci. Rep.* **6**, 33028; doi: 10.1038/srep33028 (2016).

## Supplementary Material

Supplementary Information

## References

[b1] Zhu J. *et al.* Comparative genomics search for losses of long-established genes on the human lineage. Plos Comput Biol 3, e247, doi: 10.1371/journal.pcbi.0030247 (2007). 18085818 PMC2134963

[b2] Morris J. J., Lenski R. E. & Zinser E. R. The Black Queen Hypothesis: evolution of dependencies through adaptive gene loss. MBio 3, doi: 10.1128/mBio.00036-12 (2012). PMC3315703 22448042

[b3] Hendrickson R. C., Wang C., Hatcher E. L. & Lefkowitz E. J. Orthopoxvirus genome evolution: the role of gene loss. Viruses 2, 1933–1967, doi: 10.3390/v2091933 (2010). 21994715 PMC3185746

[b4] Sabeti P. C. *et al.* Positive natural selection in the human lineage. Science 312, 1614–1620, doi: 10.1126/science.1124309 (2006). 16778047

[b5] Kreitman M. Methods to detect selection in populations with applications to the human. Annu Rev Genomics Hum Genet 1, 539–559, doi: 10.1146/annurev.genom.1.1.539 (2000). 11701640

[b6] Bustamante C. D., Wakeley J., Sawyer S. & Hartl D. L. Directional selection and the site-frequency spectrum. Genetics 159, 1779–1788 (2001). 11779814 10.1093/genetics/159.4.1779 PMC1461920

[b7] Rector A. & Van Ranst M. Animal papillomaviruses. Virology 445, 213–223, doi: 10.1016/j.virol.2013.05.007 (2013). 23711385

[b8] Van Doorslaer K. *et al.* The Papillomavirus Episteme: a central resource for papillomavirus sequence data and analysis. Nucleic Acids Res 41, D571–D578, doi: 10.1093/nar/gks984 (2013). 23093593 PMC3531071

[b9] Klingelhutz A. J. & Roman A. Cellular transformation by human papillomaviruses: lessons learned by comparing high- and low-risk viruses. Virology 424, 77–98, doi: 10.1016/j.virol.2011.12.018 (2012). 22284986 PMC3703738

[b10] Vande Pol S. B. & Klingelhutz A. J. Papillomavirus E6 oncoproteins. Virology 445, 115–137, doi: 10.1016/j.virol.2013.04.026 (2013). 23711382 PMC3783570

[b11] Roman A. & Munger K. The papillomavirus E7 proteins. Virology 445, 138–168, doi: 10.1016/j.virol.2013.04.013 (2013). 23731972 PMC3783579

[b12] DiMaio D. & Petti L. M. The E5 proteins. Virology 445, 99–114, doi: 10.1016/j.virol.2013.05.006 (2013). 23731971 PMC3772959

[b13] Van Doorslaer K. Evolution of the papillomaviridae. Virology 445, 11–20, doi: 10.1016/j.virol.2013.05.012 (2013). 23769415

[b14] Buck C. B., Day P. M. & Trus B. L. The papillomavirus major capsid protein L1. Virology 445, 169–174, doi: 10.1016/j.virol.2013.05.038 (2013). 23800545 PMC3783536

[b15] Wang J. W. & Roden R. B. L2, the minor capsid protein of papillomavirus. Virology 445, 175–186, doi: 10.1016/j.virol.2013.04.017 (2013). 23689062 PMC3770800

[b16] Bergvall M., Melendy T. & Archambault J. The E1 proteins. Virology 445, 35–56, doi: 10.1016/j.virol.2013.07.020 (2013). 24029589 PMC3811109

[b17] McBride A. A. The papillomavirus E2 proteins. Virology 445, 57–79, doi: 10.1016/j.virol.2013.06.006 (2013). 23849793 PMC3783563

[b18] Bernard H. U. *et al.* Classification of papillomaviruses (PVs) based on 189 PV types and proposal of taxonomic amendments. Virology 401, 70–79, doi: 10.1016/j.virol.2010.02.002 (2010). 20206957 PMC3400342

[b19] de Villiers E. M., Fauquet C., Broker T. R., Bernard H. U. & zur Hausen H. Classification of papillomaviruses. Virology 324, 17–27, doi: 10.1016/j.virol.2004.03.033 (2004). 15183049

[b20] Garcia-Vallve S., Alonso A. & Bravo I. G. Papillomaviruses: different genes have different histories. Trends Microbiol 13, 514–521, doi: 10.1016/j.tim.2005.09.003 (2005). 16181783

[b21] Bravo I. G. & Alonso A. Mucosal human papillomaviruses encode four different E5 proteins whose chemistry and phylogeny correlate with malignant or benign growth. J Virol 78, 13613–13626, doi: 10.1128/JVI.78.24.13613-13626.2004 (2004). 15564472 PMC533923

[b22] Zanier K. *et al.* Structural basis for hijacking of cellular LxxLL motifs by papillomavirus E6 oncoproteins. Science 339, 694–698, doi: 10.1126/science.1229934 (2013). 23393263 PMC3899395

[b23] Van Doorslaer K. *et al.* Identification of unusual E6 and E7 proteins within avian papillomaviruses: cellular localization, biophysical characterization, and phylogenetic analysis. J Virol 83, 8759–8770, doi: 10.1128/JVI.01777-08 (2009). 19553340 PMC2738182

[b24] Herbst L. H. *et al.* Genomic characterization of two novel reptilian papillomaviruses, Chelonia mydas papillomavirus 1 and Caretta caretta papillomavirus 1. Virology 383, 131–135, doi: 10.1016/j.virol.2008.09.022 (2009). 18973915

[b25] Campo M. S. *et al.* HPV-16 E5 down-regulates expression of surface HLA class I and reduces recognition by CD8 T cells. Virology 407, 137–142, doi: 10.1016/j.virol.2010.07.044 (2010). 20813390

[b26] Rector A. *et al.* Ancient papillomavirus-host co-speciation in Felidae. Genome Biol 8, R57, doi: 10.1186/gb-2007-8-4-r57 (2007). 17430578 PMC1896010

[b27] dos Reis M. *et al.* Phylogenomic datasets provide both precision and accuracy in estimating the timescale of placental mammal phylogeny. Proc Biol Sci 279, 3491–3500, doi: 10.1098/rspb.2012.0683 (2012). 22628470 PMC3396900

[b28] Perelman P. *et al.* A molecular phylogeny of living primates. Plos Genet 7, e1001342, doi: 10.1371/journal.pgen.1001342 (2011). 21436896 PMC3060065

[b29] Chen Z., Schiffman M., Herrero R., Desalle R. & Burk R. D. Human papillomavirus (HPV) types 101 and 103 isolated from cervicovaginal cells lack an E6 open reading frame (ORF) and are related to gamma-papillomaviruses. Virology 360, 447–453, doi: 10.1016/j.virol.2006.10.022 (2007). 17125811 PMC1885239

[b30] Kumar S. & Hedges S. B. A molecular timescale for vertebrate evolution. Nature 392, 917–920, doi: 10.1038/31927 (1998). 9582070

[b31] Van Doorslaer K. & Burk R. D. Evolution of human papillomavirus carcinogenicity. Adv Virus Res 77, 41–62, doi: 10.1016/B978-0-12-385034-8.00002-8 (2010). 20951869 PMC3690501

[b32] Ameur A. *et al.* Comprehensive profiling of the vaginal microbiome in HIV positive women using massive parallel semiconductor sequencing. Sci Rep 4, 4398, doi: 10.1038/srep04398 (2014). 24637939 PMC3957130

[b33] Bottalico D. *et al.* The oral cavity contains abundant known and novel human papillomaviruses from the Betapapillomavirus and Gammapapillomavirus genera. J Infect Dis 204, 787–792, doi: 10.1093/infdis/jir383 (2011). 21844305 PMC3156102

[b34] Nobre R. J. *et al.* E7 oncoprotein of novel human papillomavirus type 108 lacking the E6 gene induces dysplasia in organotypic keratinocyte cultures. J Virol 83, 2907–2916, doi: 10.1128/JVI.02490-08 (2009). 19153227 PMC2655592

[b35] Warren C. J., Van Doorslaer K., Pandey A., Espinosa J. M. & Pyeon D. Role of the host restriction factor APOBEC3 on papillomavirus evolution. Virus Evolution 1, vev015 (2015). 27570633 10.1093/ve/vev015 PMC4999249

[b36] Mengual-Chulia B., Bedhomme S., Lafforgue G., Elena S. F. & Bravo I. G. Assessing parallel gene histories in viral genomes. BMC Evol Biol 16, 32, doi: 10.1186/s12862-016-0605-4 (2016). 26847371 PMC4743424

[b37] Katoh K., Misawa K., Kuma K. & Miyata T. MAFFT: a novel method for rapid multiple sequence alignment based on fast Fourier transform. Nucleic Acids Res 30, 3059–3066 (2002). 12136088 10.1093/nar/gkf436 PMC135756

[b38] Katoh K. & Standley D. M. MAFFT: iterative refinement and additional methods. Methods Mol Biol 1079, 131–146, doi: 10.1007/978-1-62703-646-7_8 (2014). 24170399

[b39] Katoh K. & Standley D. M. MAFFT multiple sequence alignment software version 7: improvements in performance and usability. Mol Biol Evol 30, 772–780, doi: 10.1093/molbev/mst010 (2013). 23329690 PMC3603318

[b40] Lanfear R., Calcott B., Ho S. Y. & Guindon S. Partitionfinder: combined selection of partitioning schemes and substitution models for phylogenetic analyses. Mol Biol Evol 29, 1695–1701, doi: 10.1093/molbev/mss020 (2012). 22319168

[b41] Lanave C., Preparata G., Saccone C. & Serio G. A new method for calculating evolutionary substitution rates. J Mol Evol 20, 86–93 (1984). 6429346 10.1007/BF02101990

[b42] Tavare S. Some Probabilistic and Statistical Problems in the Analysis of DNA Sequences. Lectures on Mathematics in the Life Sciences 17, 30 (1986).

[b43] Rodriguez F., Oliver J. L., Marin A. & Medina J. R. The general stochastic model of nucleotide substitution. J Theor Biol 142, 485–501 (1990). 2338834 10.1016/s0022-5193(05)80104-3

[b44] Stamatakis A. RAxML version 8: a tool for phylogenetic analysis and post-analysis of large phylogenies. Bioinformatics 30, 1312–1313, doi: 10.1093/bioinformatics/btu033 (2014). 24451623 PMC3998144

[b45] Miller M. A., Pfeiffer W. & Schwartz T. In Gateway Computing Environments Workshop (GCE) 1–8 (New Orleans, LA, 2010).

[b46] Pattengale N. D., Alipour M., Bininda-Emonds O. R., Moret B. M. & Stamatakis A. How many bootstrap replicates are necessary? J Comput Biol 17, 337–354, doi: 10.1089/cmb.2009.0179 (2010). 20377449

[b47] Drummond A. J., Suchard M. A., Xie D. & Rambaut A. Bayesian phylogenetics with BEAUti and the BEAST 1.7. Mol Biol Evol 29, 1969–1973, doi: 10.1093/molbev/mss075 (2012). 22367748 PMC3408070

[b48] Nylander J. A., Wilgenbusch J. C., Warren D. L. & Swofford D. L. AWTY (are we there yet?): a system for graphical exploration of MCMC convergence in Bayesian phylogenetics. Bioinformatics 24, 581–583, doi: 10.1093/bioinformatics/btm388 (2008). 17766271

[b49] Kyte J. & Doolittle R. F. A simple method for displaying the hydropathic character of a protein. J Mol Biol 157, 105–132 (1982). 7108955 10.1016/0022-2836(82)90515-0

[b50] Hopp T. P. & Woods K. R. Prediction of protein antigenic determinants from amino acid sequences. Proc Natl Acad Sci USA 78, 3824–3828 (1981). 6167991 10.1073/pnas.78.6.3824 PMC319665

[b51] Wilkins M. R. *et al.* Protein identification and analysis tools in the ExPASy server. Methods Mol Biol 112, 531–552 (1999). 10027275 10.1385/1-59259-584-7:531

[b52] Paradis E., Claude J. & Strimmer K. APE: Analyses of Phylogenetics and Evolution in R language. Bioinformatics 20, 289–290 (2004). 14734327 10.1093/bioinformatics/btg412

[b53] Bell M. A. & Lloyd G. T. strap: an R package for plotting phylogenies against stratigraphy and assessing their stratigraphic congruence. Palaeontology 58, 379–389, doi: 10.1111/pala.12142 (2015).

