# Supplementary material for: Molecular archeological evidence in support of the repeated loss of a papillomavirus gene
Source: Sci Rep. 2016 Sep 8;6:33028. doi: 10.1038/srep33028 (PMC5015084; doi:10.1038/srep33028)
Supplement: Supplementary Information [file srep33028-s1.pdf]

Legends to the supplemental materials

Supplemental Table 1

Details of viral genomes used in the phylogenetic tree in Figure 1.

Supplemental Table 2

The reported estimates for the Bayesian dating analysis. Node numbers correspond to figure 2.

Supplemental Figure 1

High resolution, base-pair level version of Figure 3 A.

Supplemental table 1

Virus	Host Species	Viral Species Name $	Reference	Genbank ID
HPV4	Homo sapiens	Gammapapillomavirus 1	Egawa et al., 1993	X70827
HPV48	Homo sapiens	Gammapapillomavirus 2	Muller et al., 1989	U31789
HPV50	Homo sapiens	Gammapapillomavirus 3	Favre et al., 1989	U31790
HPV60	Homo sapiens	Gammapapillomavirus 4	Matsukura et al., 1992	U31792
HPV65	Homo sapiens	Gammapapillomavirus 1	Egawa et al., 1993	X70829
HPV88	Homo sapiens	Gammapapillomavirus 5	Kullander et al., 2008	EF467176
HPV95	Homo sapiens	Gammapapillomavirus 1	Egawa et al., Unpublished	AJ620210
HPV101	Homo sapiens	Gammapapillomavirus 6	Chen et al., 2007	DQ080081
HPV103	Homo sapiens	Gammapapillomavirus 6	Chen et al., 2007	DQ080078
HPV108	Homo sapiens	Gammapapillomavirus 6	Nobre et al., 2009	FM212639
HPV109	Homo sapiens	Gammapapillomavirus 7	Ekstrom et al., 2010	EU541441
HPV112	Homo sapiens	Gammapapillomavirus 8	Ekstrom et al., 2010	EU541442
HPV116	Homo sapiens	Gammapapillomavirus 9	Li et al., 2009	FJ804072
HPV119	Homo sapiens	Gammapapillomavirus 8	Bernard et al., 2010	GQ845441
HPV121	Homo sapiens	Gammapapillomavirus 10	Bernard et al., 2010	GQ845443
HPV123	Homo sapiens	Gammapapillomavirus 7	Bernard et al., 2010	GQ845445
HPV126	Homo sapiens	Gammapapillomavirus 11	Egawa et al., pres	AB646346
HPV127	Homo sapiens	Gammapapillomavirus 12	Schowalter et al., 2010	HM011570
HPV128	Homo sapiens	Gammapapillomavirus 13	Koehler et al., 2011	GU225708
HPV129	Homo sapiens	Gammapapillomavirus 9	Koehler et al., 2011	GU233853
HPV130	Homo sapiens	Gammapapillomavirus 10	Koehler et al., 2011	GU117630
HPV131	Homo sapiens	Gammapapillomavirus 14	Koehler et al., 2011	GU117631
HPV132	Homo sapiens	Gammapapillomavirus 12	Koehler et al., 2011	GU117632
HPV133	Homo sapiens	Gammapapillomavirus 10	Koehler et al., 2011	GU117633
HPV134	Homo sapiens	Gammapapillomavirus 7	Koehler et al., 2011	GU117634
HPV135	Homo sapiens	Gammapapillomavirus 15	Bottalico et al., 2011	HM999987
HPV136	Homo sapiens	Gammapapillomavirus 11	Bottalico et al., 2011	HM999988

HPV137	Homo sapiens	Gammapapillomavirus 16	Bottalico et al., 2011	HM999989
HPV138	Homo sapiens	Gammapapillomavirus 7	Chen et al., Unpublished	HM999990
HPV139	Homo sapiens	Gammapapillomavirus 7	Chen et al., Unpublished	HM999991
HPV140	Homo sapiens	Gammapapillomavirus 11	Bottalico et al., 2011	HM999992
HPV141	Homo sapiens	Gammapapillomavirus 11	Bottalico et al., 2011	HM999993
HPV142	Homo sapiens	Gammapapillomavirus 10	Chen et al., Unpublished	HM999994
HPV144	Homo sapiens	Gammapapillomavirus 17	Bottalico et al., 2011	HM999996
HPV146	Homo sapiens	Gammapapillomavirus 15	Chen et al., Unpublished	HM999998
HPV147	Homo sapiens	Gammapapillomavirus 8	Chen et al., Unpublished	HM999999
HPV148	Homo sapiens	Gammapapillomavirus 12	Koehler et al., 2011	GU129016
HPV149	Homo sapiens	Gammapapillomavirus 7	Koehler et al., 2011	GU117629
HPV153	Homo sapiens	Gammapapillomavirus 13	Sturegard et al., Unpublished	JN171845
HPV154	Homo sapiens	Gammapapillomavirus 11	Ure and Forslund, Unpublished	JN211193
HPV155	Homo sapiens	Gammapapillomavirus 7	Ekstrom et al., 2011	JF906559
HPV156	Homo sapiens	Gammapapillomavirus 18	Chouhy et al., 2013	JX429973
HPV161	Homo sapiens	Gammapapillomavirus 19	Li et al., 2012	JX413109
HPV162	Homo sapiens	Gammapapillomavirus 19	Li et al., 2012	JX413108
HPV163	Homo sapiens	Gammapapillomavirus 20	Li et al., 2012	JX413107
HPV164	Homo sapiens	Gammapapillomavirus 8	Li et al., 2012	JX413106
HPV165	Homo sapiens	Gammapapillomavirus 12	Li et al., 2012	JX444072
HPV166	Homo sapiens	Gammapapillomavirus 19	Li et al., 2012	JX413104
HPV167	Homo sapiens	Gammapapillomavirus 21	Li et al., 2013	KC862318
HPV168	Homo sapiens	Gammapapillomavirus 8	Li et al., 2013	KC862317
HPV169	Homo sapiens	Gammapapillomavirus 11	Li et al., 2012	JX413105
HPV170	Homo sapiens	Gammapapillomavirus 7	Li et al., 2012	JX413110
HPV171	Homo sapiens	Gammapapillomavirus 11	Martin et al., 2013	KF006398
HPV172	Homo sapiens	Gammapapillomavirus 22	Martin et al., 2013	KF006399
HPV173	Homo sapiens	Gammapapillomavirus 1	Martin et al., 2013	KF006400
HPV175	Homo sapiens	Gammapapillomavirus 23	Johansson et al., 2013	KC108721

HPV178	Homo sapiens	Gammapapillomavirus 24	Johansson and Forslund, 2014	KJ130020
HPV179	Homo sapiens	Gammapapillomavirus 15	Kocjan et al., Unpublished	HG421739
HPV180	Homo sapiens	Gammapapillomavirus 10	Johansson et al., 2013	KC108722
HPV184	Homo sapiens	Gammapapillomavirus 25	Kocjan et al., Unpublished	HG530535
HPV197	Homo sapiens	Gammapapillomavirus 24	Arroyo et al., Unpublished	KM085343
HPV199	Homo sapiens	Gammapapillomavirus 12	Ostrbenk et al., Unpublished	KJ913662
HPV-mCG2*	Homo sapiens	Gammapapillomavirus	Sauvage et al., Unpublished	JF966378
HPV-mCG3*	Homo sapiens	Gammapapillomavirus	Sauvage et al., Unpublished	JF966379
HPV-mCH2*	Homo sapiens	Gammapapillomavirus	Yu et al., Unpublished	KF791917
HPV-mFD1*	Homo sapiens	Gammapapillomavirus	Sauvage et al., Unpublished	JF966375
HPV-mFD2*	Homo sapiens	Gammapapillomavirus	Sauvage et al., Unpublished	JF966376
HPV-mFI864*	Homo sapiens	Gammapapillomavirus	Phan et al., 2013	KC311731
HPV-mFS1*	Homo sapiens	Gammapapillomavirus	Sauvage et al., Unpublished	JF966373
HPV-mKC5*	Homo sapiens	Gammapapillomavirus	Li et al., 2012	JX444073
HPV-mKN1*	Homo sapiens	Gammapapillomavirus	Sauvage et al., Unpublished	JF966371
HPV-mKN2*	Homo sapiens	Gammapapillomavirus	Sauvage et al., Unpublished	JF966372
HPV-mKN3*	Homo sapiens	Gammapapillomavirus	Sauvage et al., Unpublished	JF966374
HPV-mL55*	Homo sapiens	Gammapapillomavirus	Yu et al., 2013	KF482069
HPV-mSD2*	Homo sapiens	Gammapapillomavirus	Mokili et al., 2013	KC113191
CPV2	Canis familiaris	Taupapillomavirus 1	Yuan et al., 2007	AY722648

* These non-reference genomes have not been officicially recognized by the Papillomavirus reference center.
$ Papillomavirus classification is performed by the International committee for the taxonomy of viruses. Viruses without a trailing number have not yet been officially designated. The genus classification should be considered preliminary.

Supplemental table 2

node	posterior	height (95% HPD)	node	posterior	height (95% HPD)	node	posterior	height (95% HPD)	node	posterior	height (95% HPD)
1	1.00	60.64 (50.55 - 72.74)	20	1.00	25.09 (21.04 - 29.79)	39	1.00	31.00 (26.11 - 36.62)	58	1.00	19.32 (16.08 - 23.01)
2	1.00	57.28 (48.51 - 67.63)	21	1.00	14.90 (11.82 - 18.35)	40	1.00	25.08 (21.02 - 29.82)	59	1.00	11.68 (9.32 - 14.34)
3	1.00	23.24 (18.56 - 28.61)	22	1.00	23.06 (19.25 - 27.44)	41	1.00	22.32 (18.58 - 26.56)	60	1.00	12.47 (10.04 - 15.27)
4	1.00	12.49 (9.54 - 15.67)	23	1.00	18.99 (15.75 - 22.70)	42	1.00	15.11 (12.34 - 18.21)	61	1.00	23.66 (19.68 - 28.27)
5	1.00	54.56 (46.24 - 64.30)	24	1.00	10.20 (8.05 - 12.64)	43	1.00	12.42 (9.97 - 15.15)	62	1.00	19.71 (16.13 - 23.83)
6	1.00	26.91 (22.19 - 32.48)	25	1.00	15.22 (12.35 - 18.48)	44	1.00	10.80 (8.45 - 13.52)	63	0.96	43.36 (36.77 - 51.11)
7	1.00	19.80 (16.54 - 23.61)	26	1.00	22.16 (18.23 - 26.63)	45	1.00	30.42 (25.17 - 36.50)	64	0.80	41.07 (34.71 - 48.43)
8	1.00	16.69 (13.59 - 20.24)	27	1.00	18.67 (15.17 - 22.73)	46	1.00	39.26 (33.32 - 46.25)	65	1.00	16.67 (13.59 - 20.10)
9	1.00	17.19 (14.30 - 20.54)	28	1.00	33.40 (28.01 - 39.59)	47	1.00	31.93 (26.93 - 37.94)	66	1.00	10.41 (8.12 - 13.06)
10	1.00	15.33 (12.69 - 18.38)	29	1.00	18.30 (15.17 - 21.93)	48	1.00	18.84 (15.05 - 23.06)	67	1.00	10.50 (8.27 - 13.01)
11	1.00	10.80 (8.64 - 13.32)	30	1.00	16.01 (13.23 - 19.22)	49	1.00	21.13 (17.44 - 25.41)	68	1.00	25.51 (20.92 - 30.74)
12	1.00	10.41 (8.33 - 12.78)	31	1.00	13.00 (10.63 - 15.71)	50	1.00	17.88 (14.66 - 21.67)	69	1.00	21.48 (17.52 - 26.10)
13	1.00	48.94 (41.65 - 57.70)	32	1.00	9.24 (7.37 - 11.38)	51	1.00	12.43 (9.89 - 15.35)	70	1.00	10.62 (8.57 - 12.99)
14	1.00	45.25 (38.55 - 53.38)	33	1.00	37.29 (31.40 - 44.14)	52	1.00	33.72 (28.48 - 39.78)	71	1.00	6.67 (5.18 - 8.30)
15	1.00	43.16 (36.70 - 50.82)	34	1.00	31.86 (26.50 - 37.87)	53	1.00	30.11 (25.41 - 35.57)	72	1.00	16.66 (13.72 - 20.03)
16	1.00	41.83 (35.57 - 49.30)	35	1.00	15.00 (12.04 - 18.32)	54	1.00	24.48 (20.54 - 28.93)	73	1.00	14.26 (11.71 - 17.22)
17	0.99	40.45 (34.38 - 47.75)	36	1.00	7.91 (6.18 - 9.96)	55	0.60	22.70 (19.05 - 26.87)	74	1.00	10.09 (8.07 - 12.42)
18	1.00	31.36 (26.42 - 37.08)	37	1.00	41.24 (35.14 - 48.67)	56	1.00	13.76 (11.07 - 16.82)	75	1.00	6.53 (5.04 - 8.18)
19	1.00	27.23 (22.83 - 32.25)	38	1.00	36.89 (31.18 - 43.46)	57	1.00	3.16 (2.41 - 3.99)			

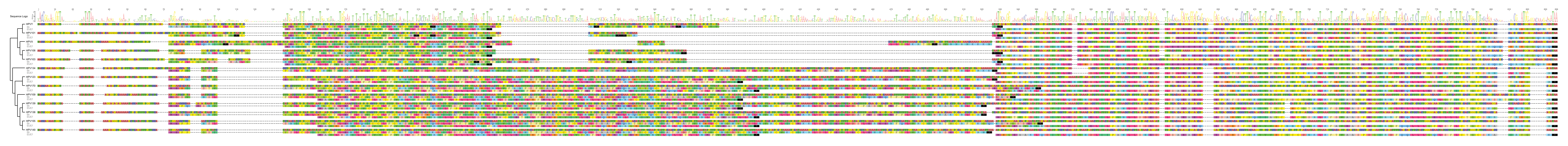
Sequence Logo
